# Pd@Ag Nanosheets in Combination with Amphotericin B Exert a Potent Anti-Cryptococcal Fungicidal Effect

**DOI:** 10.1371/journal.pone.0157000

**Published:** 2016-06-07

**Authors:** Chao Zhang, Mei Chen, Guizhen Wang, Wei Fang, Chen Ye, Hanhua Hu, Zhenzong Fa, Jiu Yi, Wan-qing Liao

**Affiliations:** 1 Shanghai Key Laboratory of Molecular Medical Mycology, Shanghai Institute of Medical Mycology, Second Military Medical University, Shanghai, China; 2 PLA Key Laboratory of Mycosis, Department of Dermatology and Venereology, Changzheng Hospital, Shanghai, China; 3 State Key Laboratory for Physical Chemistry of Solid Surfaces, Collaborative Innovation Centre of Chemistry for Energy Materials and Department of Chemistry, College of Chemistry and Chemical Engineering, Xiamen University, Xiamen, China; 4 ICU department, Urumuqi Army General Hospital, Urumqi, Xinjiang, China; 5 Department of Urology, Changhai Hospital, Second Military Medical University, Shanghai, China; 6 UEM department, Xinjiang Medical University, Urumqi, Xinjiang, China; University of Minnesota, UNITED STATES

## Abstract

Silver nanoparticles have received considerable interest as new “nanoantibiotics” with the potential to kill drug-resistant microorganisms. Recently, a class of new core-shell nanostructures, Pd@Ag nanosheets (Pd@Ag NSs), were created using deposition techniques and demonstrated excellent inhibitory effects on various bacteria in vitro. In this study, we evaluated the antifungal activity of Pd@Ag NSs against common invasive fungal pathogens. Among these organisms, *Cryptococcus neoformans* complex species was most susceptible to Pd@Ag NSs, which exhibited potent antifungal activity against various molecular types or sources of cryptococcal strains including fluconazole-resistant isolates. The anticryptococcal activity of Pd@Ag NSs was significantly greater than fluconazole and similar to that of amphotericin B (AmB). At relatively high concentrations, Pd@Ag NSs exhibited fungicidal activity against *Cryptococcus spp*., which can likely be attributed to the disruption of cell integrity, intracellular protein synthesis, and energy metabolism. Intriguingly, Pd@Ag NSs also exhibited strong synergistic anti-cryptococcal fungicidal effects at low concentrations in combination with AmB but exhibited much better safety in erythrocytes than AmB, even at the minimal fungicidal concentration. Therefore, Pd@Ag NSs may be a promising adjunctive agent for treating cryptococcosis, and further investigation for clinical applications is required.

## Introduction

*Cryptococcus neoformans* complex species (including *C*. *neoformans* and *C*. *gattii*) is one of the most important invasive fungal pathogens worldwide and can cause life-threatening meningoencephalitis in both immunocompromised and immunocompetent populations [1, 2]. Due to the HIV epidemic and increasing application of immunosuppressive therapies, cryptococcosis has become a major burden to health care globally over the past two decades [3]. In 2008, Park et al. once estimated that approximately 1 million new cases of cryptococcal meningitis are identified annually, which gives rise to over 600,000 deaths each year [4]. Currently, the mortality rate remains at about 20% even with prompt treatment, while it is much higher (30%)in resource-limited areas such as sub-Saharan Africa [4–7]. This poor outcome is largely attributed to factors such as the limited number of available antifungal agents, their significant toxic effects, and the emergence of resistant fungal strains [8–10]. Amphotericin B (AmB), the cornerstone therapy of *Cryptococcus spp*., has several dose-related and infusion-related adverse effects such as nephrotoxicity, haemolytic anaemia, fever, chills, and vomit [11], which have limited its clinical utility, especially in debilitated patients. In addition, the increase in cryptococcal isolates resistant to fluconazole (FLZ) [12–15] generates another new therapeutic challenge. For example, a recent global survey of approximately 3,000 cryptococcal isolates indicated a progressive increase of FLZ-resistance from 7.3% in 1997 to 11.7% in 2007 [16]. Therefore, there is an urgent need to develop novel antimicrobial agents and/or new methods for effective anticryptococcal treatment.

Due to great advances in nanotechnology in the past decade, silver nanoparticles and silver-based materials have received increased attention as promising antimicrobial agents [17–19]. At very low concentrations, silver nanomaterials (nano-Ag) exhibit potent bactericidal activity against both Gram-positive and Gram-negative bacteria but no significant toxicity in human cells [18, 20–23]. Their antimicrobial effects have been shown to be closely associated with intracellular reactive oxygen species (ROS) accumulation, disruption of biofilm formation, and damage to the main components of micro-organisms such as the cell membrane, DNA, and intracellular proteins [24–27]. Recently, silver nanomaterials and their derivatives have also been reported to exert antifungal activity against *Candida spp*., dermatophytes, and ocular pathogenic filamentous fungi [28–32].

In 2011, Huang’s team first synthesized a class of new core-shell nanostructures, Pd@Ag nanosheets (Pd@Ag NSs), by coating palladium nanosheets with Ag [33]. Compared to pure silver nanostructures, Pd@Ag NSs displayed fascinating physical and chemical properties such as uniform sizes and shapes, tuneable optical properties, enhanced photothermal stability, and excellent biocompatibility [33–35]. Recently, these novel nanoparticles have been shown to be highly efficient for killing cancer cells and antibiotic-resistant bacteria [35–39]. Whether Pd@Ag NSs have a similar antifungal effect against common fungal pathogens such as *Cryptococcus spp*. remains to be determined.

In the present study, Pd@Ag NSs with different sizes were synthesized and tested for antifungal activity against a panel of invasive fungal pathogens. Among these organisms, cryptococcal strains, and even FLZ resistance isolates, were most susceptible to Pd@Ag-80nm nanosheets. Pd@Ag NSs at relatively high concentrations exhibited fungicidal activity against *Cryptococcus spp*., which is associated with the disruption of cell integrity and the disturbance of intracellular protein and energy metabolism. Interestingly, in combination with AmB, but not FLZ, Pd@Ag NSs at low concentrations displayed a potent effect against *C*. *neoformans*. Moreover, Pd@Ag NSs had much less potential toxicity on host erythrocytes than AmB. Our study paves the way for future development of Pd@Ag NSs application as an adjunct therapeutic agent for cryptococcosis.

## Results

### Synthesis and characterization of Pd@Ag Nanosheets

**Fig 1:**
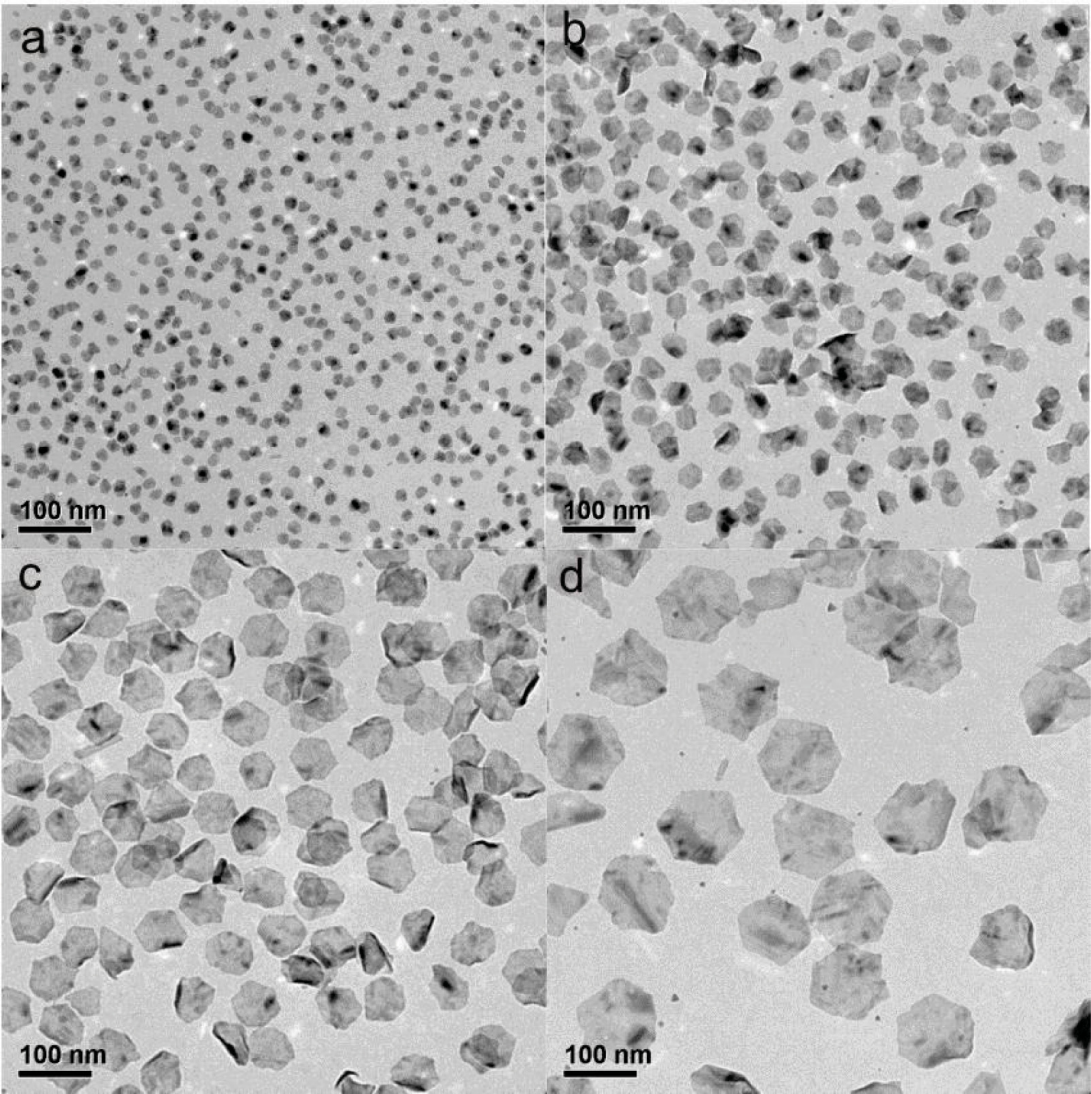
Representative large-area TEM images of Pd@Ag nanosheets with different sizes. a. 11 nm; b. 30 nm; c. 80 nm; d. 120 nm. Scale bar = 100 nm.

Pd@Ag nanosheets were synthesized by a hydride-induced-reduction strategy, utilizing Pd NSs as seeds according to a previously reported method with slight modifications [33, 39]. As shown in Fig 1, the synthesized Pd@Ag nanosheets adopted a hexagonal shape similar to the Pd NSs. By changing the sizes of the Pd NSs, Pd@Ag nanosheets with sizes similar to those of the original Pd NSs seeds (approximately 11 nm, 30 nm, 80 nm, and 120 nm) could be obtained. To ensure different sized Pd@Ag nanosheets with the same Ag/Pd molar ratio, the same amount of AgNO_3_ solution was added to different-sized Pd NS solution. The Ag/Pd molar ratios of the Pd@Ag nanosheets were all approximately 6, as determined by ICP-MS. According to UV-Vis spectroscopy, the maximum optical absorption of the resulting solutions changed from 380 nm to 1300 nm as the size of Pd@Ag nanosheets increased (data not shown).

### Among common invasive fungal pathogens, Cryptococcus spp. was most susceptible to Pd@Ag NSs

We next evaluated the fungistatic activities of Pd@Ag NSs with different diameters (11–120 nm) against common invasive fungal pathogens using the standard microdilution method. The nanoparticle solutions were diluted in sterile water to final concentrations ranging from 0.125 μg/mL to 64 μg/mL, while FLZ and AmB were utilized as control agents. As shown in Table 1, the Pd@Ag NSs inhibited all of the tested fungal strains at different concentrations, and the MIC values for Pd@Ag NSs were generally lower than those for FLZ but higher than those for AmB in most fungal pathogens. *Cryptococcus spp*. was most susceptible to the nanoparticle. The lowest MIC values were obtained at a concentration of 0.5–1 μg/mL, which was very similar to that of AmB (0.125–0.5 μg/mL). Of the *Candida spp*., *C*. *krusei* displayed higher susceptibilities to Pd@Ag NSs (1 μg/mL), while the other species, especially *C*. *glabrata*, were relatively resistant (8–16 μg/mL). Similar drug sensitivities were also observed in common mold pathogens. The growth of *Aspergillus fumigatus* and *Rhizopus oryzae* were inhibited at MICs of 4–8 μg/mL and 8–16 μg/mL, respectively. With regard to the particle sizes, the MIC values of Pd@Ag NSs for each pathogen were very similar. In *Cryptococcus spp*., the 120 nm Pd@Ag NSs (MIC = 0.5–1 μg/mL) displayed a slight disadvantage in fungistatic activity compared to the nanoparticles of other sizes. Among other pathogens, however, 80 nm Pd@Ag NSs generally exhibited slightly better antifungal effects than the other nano-Ag. The results suggested that Pd@Ag NSs exhibited stronger fungistatic activities on *Cryptococcus spp*. than on other invasive fungal pathogens.

**Table 1:** Fungistatic activity of Pd@Ag nanosheets with different sizes against common invasive fungal pathogens.

		MIC(μg/mL)					
No.	Fungal strains	Pd@Ag NSs				FLZ	AmB
		11nm	30nm	80nm	120nm		
1	*Cryptococcus neoformans* H99	0.5	0.5	0.5	1	16	0.125
2	*Cryptococcus gattii* R265	0.5	0.5	0.5	0.5	16	0.5
3	*Candida albicans* ATCC14053	16	8	4	8	>64	0.125
4	*Candida albicans* SC5314	16	16	4	8	>64	0.25
5	*Candida glabrata* ATCC28226	16	16	8	16	>64	0.5
6	*Candida krusei* ATCC2159	1	1	1	1	32	0.5
7	*Candida tropicalis* ATCC20026	8	8	4	8	1	0.125
8	*Candida parapsilosis* ATCC20406	8	8	4	8	8	0.125
9	*Candida parapsilosis* ATCC22019	8	4	2	4	1	0.25
10	*Aspergillus fumigatus* ATCC MYA-3627	8	8	4	8	>64	1
11	*Rhizopus oryzae*	8	16	8	16	16	0.5

The in vitro fungistatic activity was determined for yeasts and filamentous fungi according to the M27-A3 CLSI document and the M38-A2 guidelines, respectively. The MIC for amphotericin B (AmB) and Pd@Ag NSs was defined as 100% growth inhibition compared to the drug-free control well, while the MIC for fluconazole (FLZ) was 50% growth inhibition.

### Pd@Ag 80nm nanosheets at lower concentrations displayed good fungistatic activity for all cryptococcal strains tested

Among all of the Pd@Ag nanoparticles, the 80 nm nanosheets displayed optimal antifungal effects on all of the invasive fungal pathogens, especially *C*. *neoformans* complex species. Therefore, we decided to focus on the anticryptococcal effects of Pd@Ag NSs 80nm. To determine whether the susceptibility to Pd@Ag NSs is a general trait of Cryptococcus, 44 *C*. *neoformans* or *C*. *gattii* isolates with different molecular types or from different sources were utilized to test the susceptibility to this nanoagent, while FLZ and AmB served as the controls. The MIC values of 80 nm Pd@Ag NSs for both *C*. *neoformans* and *C*. *gattii* strains were stable, ranging from 0.25 to 2.0 μg/mL (Table 2), suggesting that the antifungal activity might be not associated with the molecular types or sources of fungal strains. Furthermore, the 80 nm Pd@Ag NSs generally exhibited better antifungal effects than FLZ (MICs, 8.0–64 μg/mL) and fungistatic effects similar to those of AmB (MICs, 0.25–1.0 μg/mL) for all the strains. Two FLZ-resistant strains, *C*. *neoformans* SCZ 50098 and SCZ 50112 (MICs>64 μg/mL), were susceptible to the nanoparticles and showed much lower MICs, 2 and 0.5 μg/mL, respectively.

**Table 2:** MICs of 80 nm Pd@Ag NSs against 44 cryptococcal isolates with different molecular types or from different sources compared to fluconazole and amphotericin B.

	MIC(μg/mL)			
Strain	Pd@Ag (80nm)	FLZ	AmB	Annotation
***C*. *neoformans***				
WM148	0.5	32	0.5	Cn VNI
WM626	2	2	0.5	Cn VNII
WM628	0.25	8	0.5	Cn VNIII
WM629	0.25	4	0.5	Cn VNIV
SCZ50097	1	16	1	Clinical (Cn VNI)
SCZ 50098	2	>64	4	Clinical (Cn VNI)
SCZ 50099	1	16	1	Clinical (Cn VNI)
SCZ 50100	2	8	0.5	Clinical (Cn VNI)
SCZ 50101	1	16	0.5	Clinical (Cn VNI)
SCZ 50102	1	16	0.5	Clinical (Cn VNI)
SCZ 50103	1	8	0.5	Clinical (Cn VNI)
SCZ 50104	1	8	0.5	Clinical (Cn VNI)
SCZ 50105	2	16	0.5	Clinical (Cn VNI)
SCZ 50106	2	8	1	Clinical (Cn VNI)
SCZ 50107	1	16	0.5	Clinical (Cn VNI)
SCZ 50108	0.5	16	1	Clinical (Cn VNI)
SCZ 50109	2	16	0.5	Clinical (Cn VNI)
SCZ 50110	1	4	0.5	Clinical (Cn VNI)
SCZ 50111	1	8	1	Clinical (Cn VNI)
SCZ 50112	0.5	>64	0.5	Clinical (Cn VNI)
SCZ 50124	1	8	1	Environmental (Cn VNI)
SCZ 50125	2	16	0.5	Environmental (Cn VNI)
SCZ 50126	2	32	0.5	Environmental (Cn VNI)
SCZ 50127	1	16	1	Environmental (Cn VNI)
**Range**	**0.25–2**	**2->64**	**0.5–4**	
***C*. *gattii***				
WM179	2	8	0.5	Cg VGI
WM178	0.5	16	1	Cg VGII
WM161	0.25	8	0.5	Cg VGIII
WM779	1	32	0.5	Cg VGIV
EJB76	1	64	1	Animal (Cg VGIIb)
EJB52	1	16	0.5	Animal (Cg VGIIc)
CBS10090	1	64	1	Clinical (Cg VGII)
EJB21	1	32	0.5	Clinical (Cg VGIIa)
ICB107	1	16	0.25	Clinical (Cg VGIIa)
NIH444	1	16	1	Clinical (Cg VGIIa)
A6MR38	1	32	0.5	Clinical (Cg VGIIc)
EJB11	1	64	1	Clinical (Cg VGIIIa)
NIH112	1	16	0.5	Clinical (Cg VGIIIb)
ICB108	1	16	0.25	Clinical (Cg VGIIIb)
MMRL2872	1	32	0.5	Clinical (Cg VGIV)
MMRL3019	1	16	0.5	Clinical (Cg VGIV)
WM276	0.5	32	0.5	Environmental (Cg VGI)
E555	0.5	16	0.25	Environmental (Cg VGI)
CBS7750	0.5	16	0.5	Environmental (Cg VGIIa)
Ram2	1	32	0.5	Environmental (Cg VGIIb)
**Range**	**0.25–2**	**8–64**	**0.25–1**	

Cn, *C*. *neoformans*; Cg, *C*. *gattii*. VNI-VNIV represent the molecular types of *C*. *neoformans*, and VGI-VGIV represent the molecular types of *C*. *gattii*.

### Pd@Ag NSs and AmB at lower concentrations showed a synergetic fungicidal effect against *Cryptococcus spp*.

We further evaluated the fungicidal effect of Pd@Ag 80 nm Nanosheets on *C*. *neoformans* complex species. Ten cryptococcal strains, including all molecular types, were used to determine the MFC of Pd@Ag NSs. Cells obtained from the microdilution assay after incubation with Pd@Ag NSs at various concentrations were also plated on drug-free medium to determine the CFU and the MFC. As shown in Table 3, the MFCs of these cryptococcal strains, ranging from 32 to 128 μg/mL, were approximately 16–64-fold the maximum MICs. These data indicated that Pd@Ag NSs alone were fungicidal at relatively high and unstable concentrations, which might be unfavourable for the clinical use of Pd@Ag NSs as a monotherapy for treating cryptococcal infections.

**Table 3:** 80 nm Pd@Ag NSs and amphotericin B exhibit a synergistic fungicidal effect.

Species	Strains	MIC(μg/mL)			FICI	MFC(μg/mL)			FFCI
		Pd@Ag	AmB	Pd@Ag/AmB		Pd@Ag	AmB	Pd@Ag/AmB	
*C*. *neoformans*	H99	0.5	0.125	0.06/0.004	0.152	32	2	4/0.004	0.127
*C*. *gattii*	R265	0.5	0.5	0.06/0.004	0.128	32	1	8/0.008	0.258
*C*. *neoformans*	WM629	0.25	0.5	0.06/0.004	0.248	32	2	8/0.004	0.252
*C*. *neoformans*	WM628	0.25	0.5	0.06/0.004	0.248	64	1	16/0.06	0.310
*C*. *neoformans*	WM148	0.5	0.5	0.06/0.004	0.128	64	1	8/0.06	0.185
*C*. *neoformans*	WM626	2	0.5	0.06/0.004	0.038	128	2	16/0.125	0.188
*C*.*gattii*	WM161	0.25	0.5	0.06/0.004	0.248	64	2	4/0.125	0.125
*C*.*gattii*	WM178	0.5	1	0.06/0.004	0.124	32	1	4/0.015	0.140
*C*.*gattii*	WM179	2	0.5	0.06/0.004	0.038	128	2	8/0.06	0.093
*C*.*gattii*	WM779	1	0.5	0.06/0.004	0.068	64	1	16/0.125	0.375

**Fig 2:**
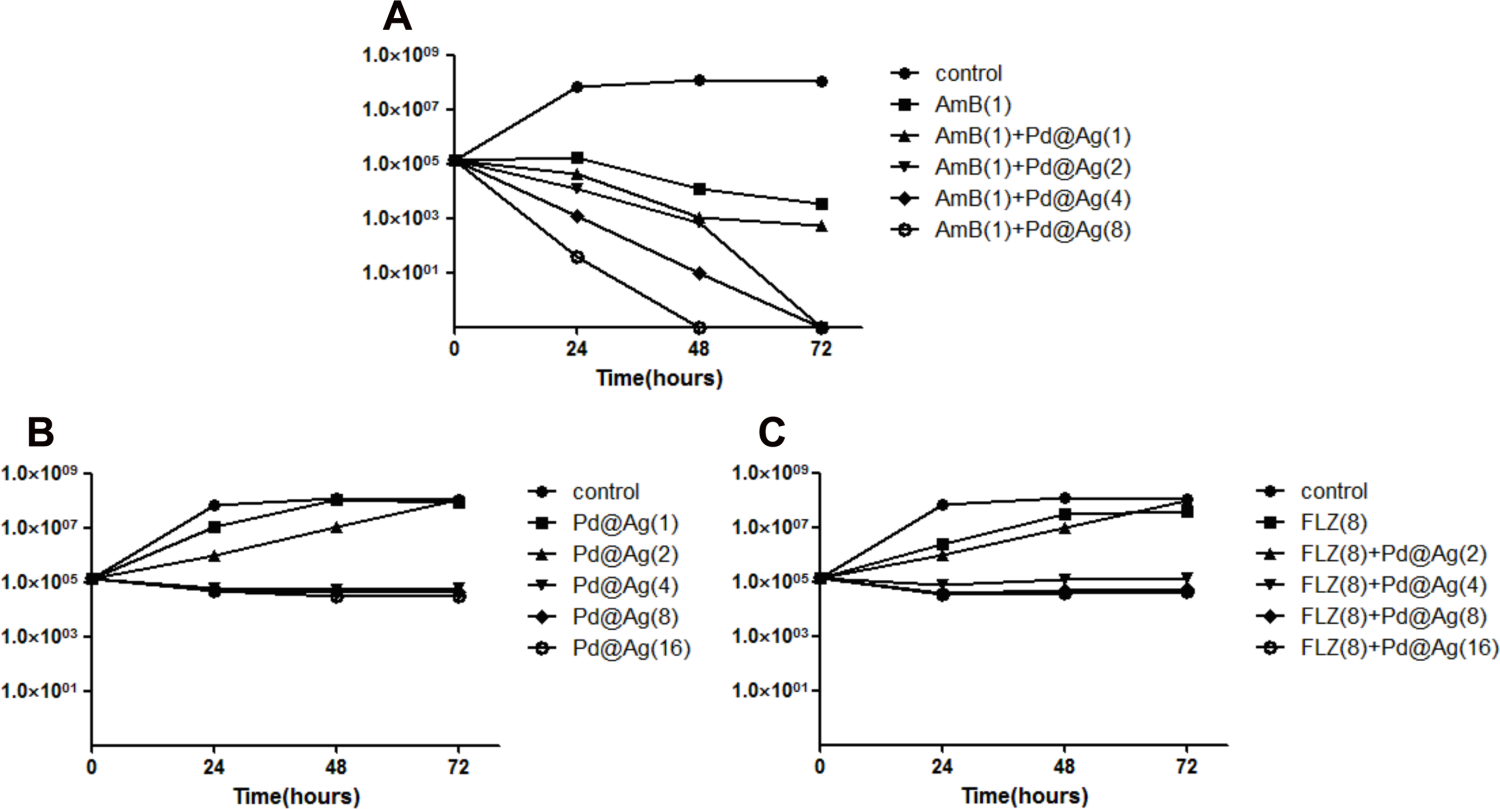
In vitro time-killing assay. The numbers of colony forming units of *C*. *neoformans* (H99) were determined after treatment with different concentrations of Pd@Ag NSs alone or combined with drugs for various periods of time. A. The Pd@Ag NSs treated group. B. The group treated with Pd@Ag NSs in combination with amphotericin B (1 μg/mL). C. The group treated with Pd@Ag NSs in combination with fluconazole (8 μg/mL). Experiments were performed in triplicate, while no drug treatment was used on the control group. The numbers in the brackets denote concentrations (μg/mL).

Therefore, combinations of Pd@Ag NSs with FLZ or AmB were also examined in a time-killing assay. *C*. *neoformans* complex species consists of eight molecular types (VN I-IV and VG I-IV), in which molecular type VN I is responsible for the majority of cryptococcosis cases (more than 90%). Thus, H99 was selected as the reference strain. Different concentrations of Pd@Ag NSs were tested alone or in combination with AmB (1 μg/mL)/FLZ (8 μg/mL). As shown in Fig 2, Pd@Ag NSs displayed a slightly inhibitory effect, even at 16 μg/mL, alone or in combination with FLZ. The presence of FLZ did not enhance the anticryptococcal effect of Pd@Ag NSs. However, the time-dependent synergistic fungicidal effect was significant for the combination of Pd@Ag NSs and AmB. The fungicidal effect was positively correlated with the concentration of Pd@Ag NSs. At 1μg/mL, Pd@Ag NSs slightly enhanced the inhibitory effect of AmB, producing a decrease in the viable cell count. At higher concentrations (e.g., 2 and 8 μg/mL), the yeast was completely killed after 48 and 72 hours cultivation, respectively. The time-dependence study confirmed that Pd@Ag NSs had synergetic fungicidal effects against *Cryptococcus spp*. with AmB but not with FLZ.

The synergistic antifungal effect was also evaluated by a microdilution assay (Table 3). Ten cryptococcal isolates were tested, including all of the molecular types. Pd@Ag NSs, even at 0.06 μg/mL, displayed a synergistic fungistatic effect when combined with AmB at 0.004 μg/mL. Furthermore, combination with Pd@Ag NSs at lower concentrations (4–16 μg/mL) significantly reduced the fungicidal concentrations of AmB (0.004–0.125 μg/mL) required for use against *C*. *neoformans* complex species. Both the FICI (0.038–0.248) and FFCI (0.093–0.375) indexes strongly supported a synergistic anticryptococcal effect of Pd@Ag NSs and AmB.

### Pd@Ag NSs-mediated damage to the morphological structure and cell membrane permeability in *C*. *neoformans*

**Fig 3:**
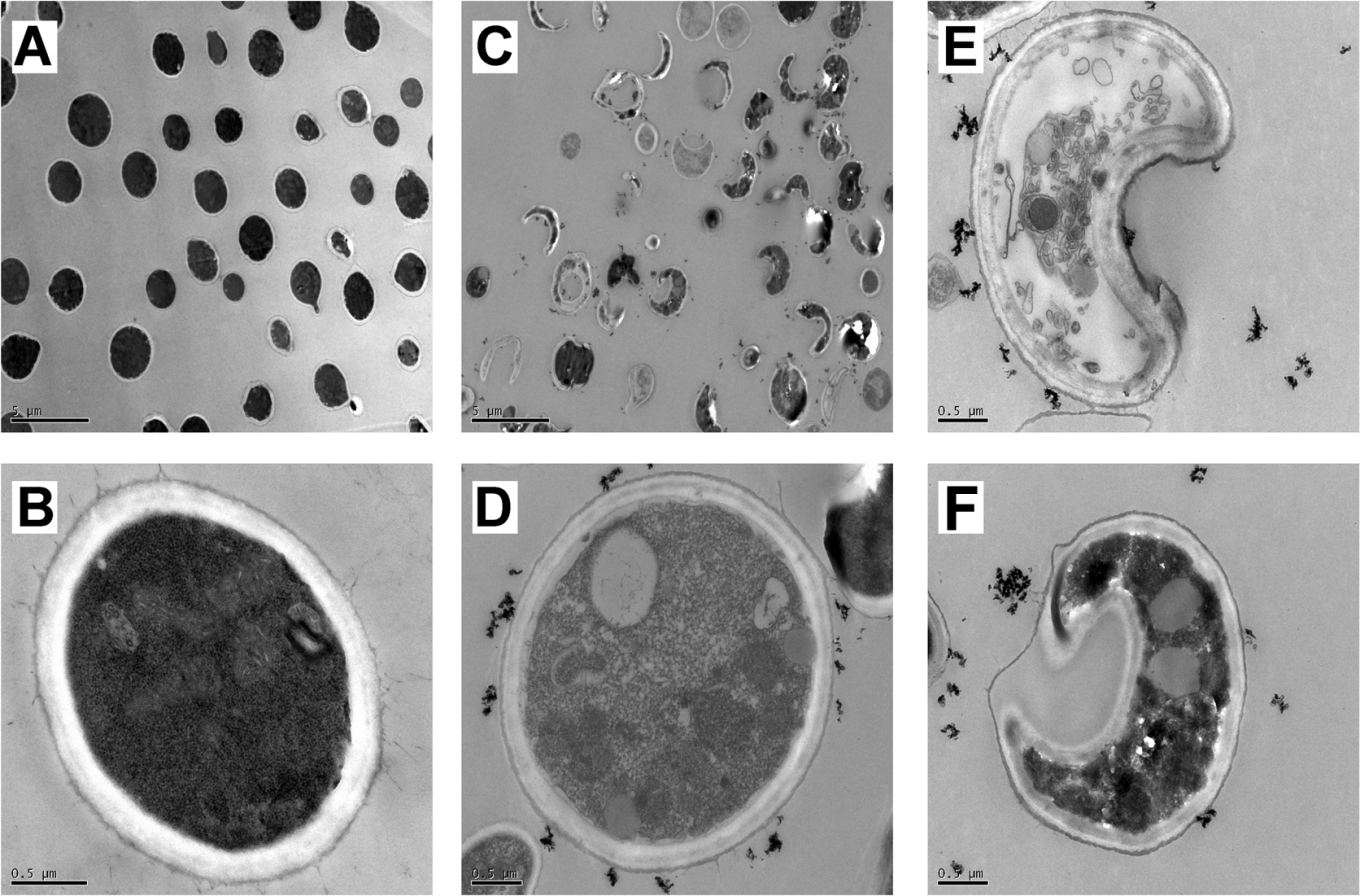
**TEM images of cryptococcal morphology before (A and B) and after (C-F) treatment with Pd@Ag NSs for 24 hours at 37°C.** Scale bars: 5 μm for A and C; 0.5 μm for B, D, E, and F.

To elucidate the possible mechanism of the effects of Pd@Ag NSs against yeast, the morphology of *C*. *neoformans* H99 was observed by transmission electron microscopy before and after the treatment with Pd@Ag NSs (16 μg/mL). The treated yeast cells exhibited remarkable morphological and structural changes compared to the untreated group (Fig 3). First, most of the treated cells appeared highly deformed with crescent shapes and were surrounded by agglomerated nanoparticles, whereas the untreated cells maintained round or ovoid shapes (Fig 3A and 3C), indicating severe damage or death of cryptococcal cells caused by Pd@Ag NSs. In addition, the structure of the cell wall in the treated group had a very loose and disordered arrangement, and obvious separation of the cell wall from the inner membrane and the outer capsule was evident in some cells (Fig 3E and 3F). Finally, prominent alterations of the intracellular structure were also observed. Some mitochondria and myelin were disfigured, and many granular ribosomes were found in the untreated cells, which displayed a high density of the cytoplasm component (Fig 3B). However, in fungal cells treated with Pd@Ag NSs, a pronounced increase in the number of vacuoles and mitochondria was evident in addition to the reduced free ribosome and cytoplasm density (Fig 3D–3F), suggesting an imbalance in protein synthesis and transportation, in addition to disrupted energy metabolism. Our data suggested that Pd@Ag NSs might disrupt the cell integrity and disturb the intracellular homeostasis of *C*. *neoformans*.

**Fig 4:**
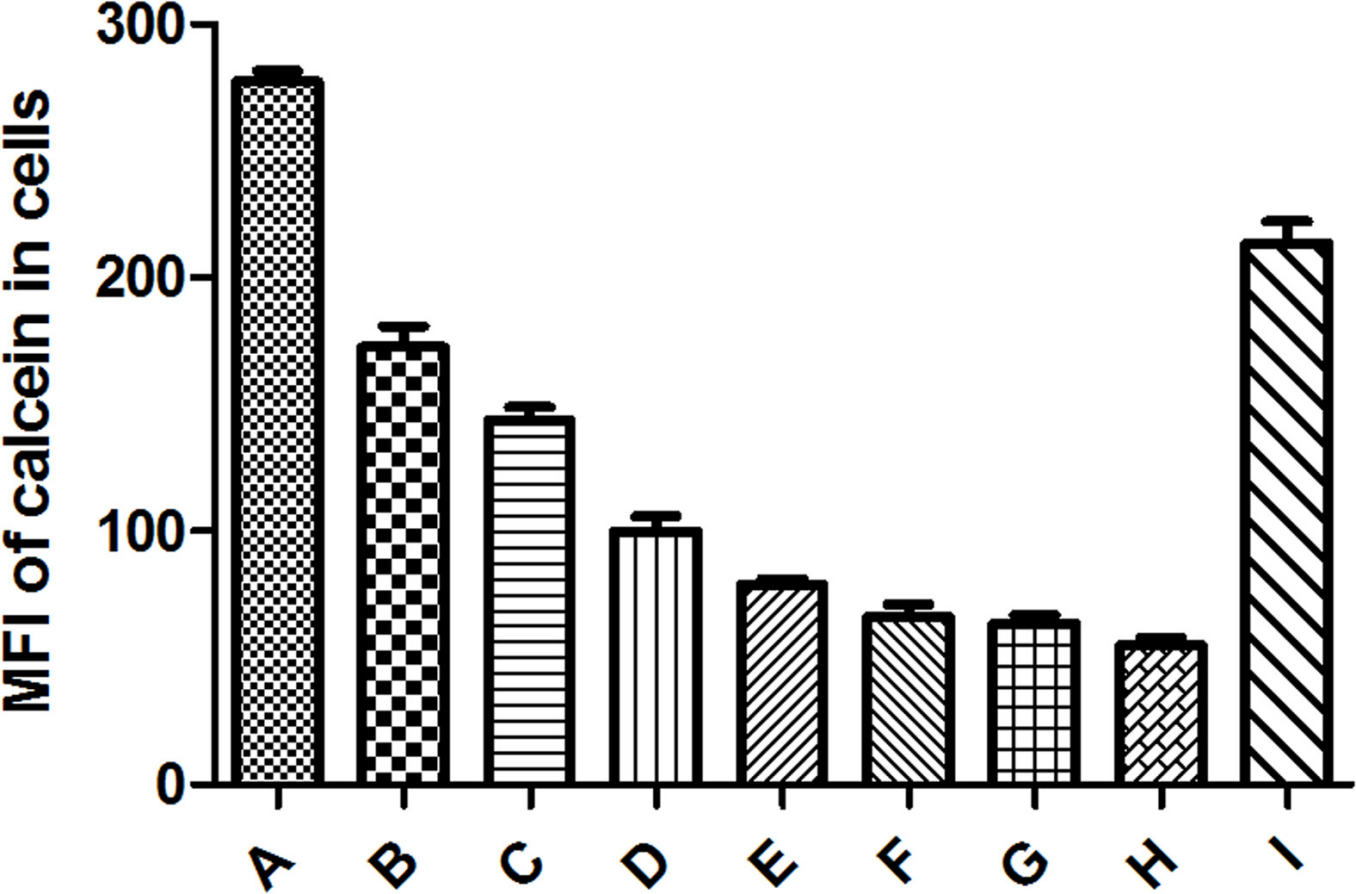
Cell membrane permeability evaluations. Cryptococcal cells (1×10^7^ cells/mL) were cultured in the presence of calcein AM for 2 h at room temperature. After washing three times, the cells were incubated with different concentrations of Pd@Ag NSs (2–128 μg/mL) for 3 h at 37°C; AmB (2 μg/mL) and PBS were utilized as the controls. The mean fluorescence intensities (MFIs) of cellular calcein in the cells were analysed by flow cytometry. Each bar represents the mean ± SD of triplicate assays. A. control; B. Pd@Ag 2 μg/mL; C. Pd@Ag 4 μg/mL; D. Pd@Ag 8 μg/mL; E. Pd@Ag 16 μg/mL; F. Pd@Ag 32 μg/mL; G. Pd@Ag 64 μg/mL; H. Pd@Ag 128 μg/mL; I. AmB 2 μg/mL.

We also assessed the effect of Pd@Ag NSs on the cell membrane permeability of *C*. *neoformans* using the non-fluorescent derivative of calcein, calcein AM. Calcein AM diffuses across membranes and is then converted into membrane-impermeable calcein by the hydrolysation of cytoplasmic esterases. After incubation with calcein AM, the cellular fluorescence of calcein was detected and quantified by flow cytometry to evaluate the effect of Pd@Ag NSs on the cell membrane permeabilization of *C*. *neoformans*. As shown in Fig 4, compared to that of the control, cellular calcein displayed an ongoing reduction from 17.5% to 80.2% with increasing concentrations of 80 nm Pd@Ag NSs (1–128 μg/mL), while only a slight reduction (23.1%) of cellular calcein was observed in the AmB group, even at the MFC (2 μg/mL). Therefore, the fungicidal effect of Pd@Ag NSs might also be associated with membrane-disrupting activities.

### Pd@Ag NSs exhibited better safety in erythrocytes than AmB

**Fig 5:**
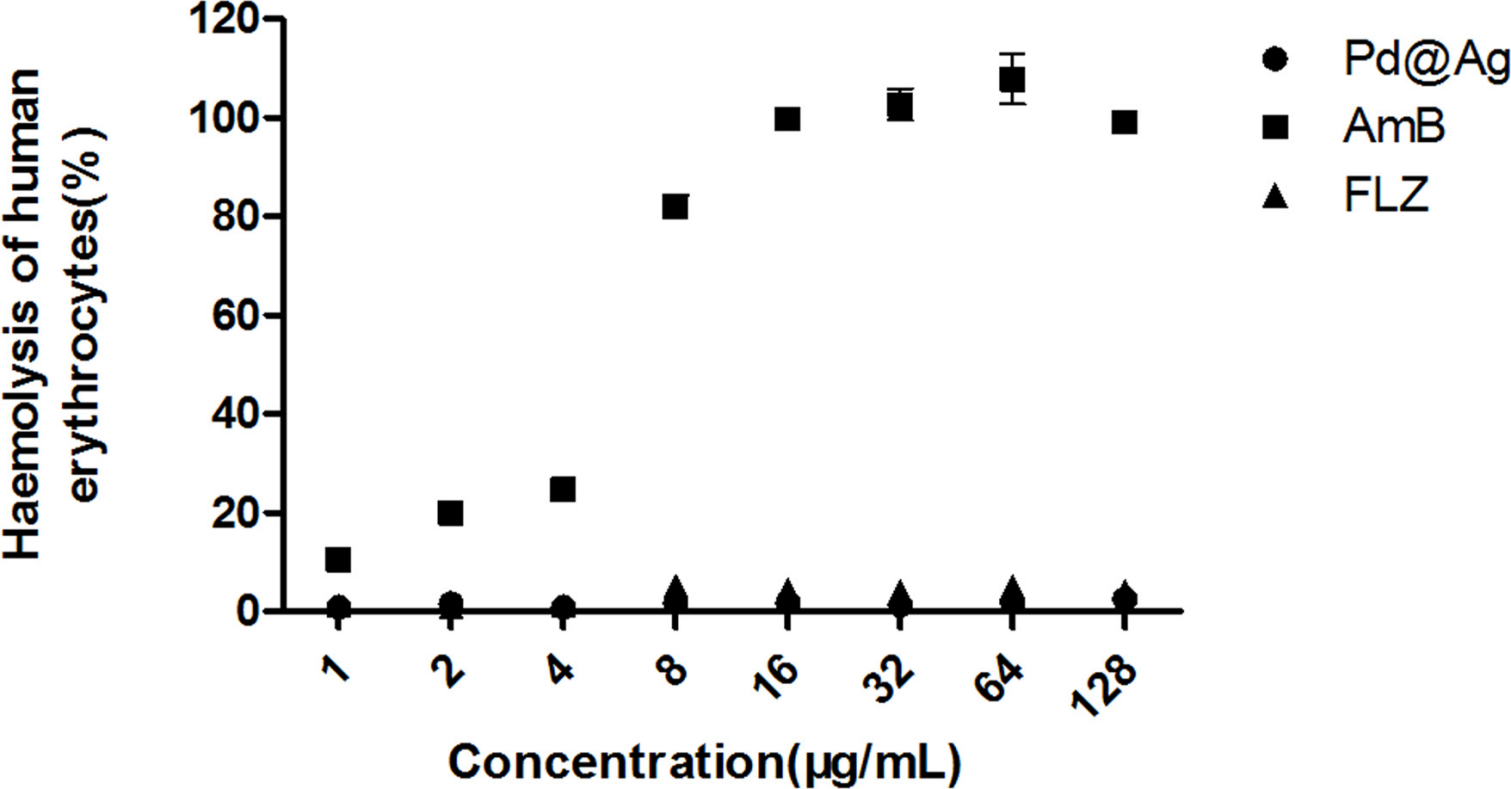
Haemolytic activity assessment of Pd@Ag NSs at different concentrations. Amphotericin B and fluconazole at the same concentrations were utilized as the controls.

Due to their membrane-damaging effects on *C*. *neoformans*, we determined whether Pd@Ag NSs had similar side effects in host cells. A haemolytic assay was performed to test the toxicity of the nanoagent at different doses on human red blood cells; AmB and FLZ were used as controls. As shown in Fig 5, AmB displayed a dose-dependent enhancement of haemolysis, and the haemolytic activity reached approximately 10.5%, even at a concentration of 1 μg/mL. However, the 80 nm Pd@Ag NSs exhibited little haemolytic activity (<3%), even at the maximum MFC (128 μg/mL), which was not statistically significant compared to FLZ. Our data indicated that Pd@Ag NSs exhibited better safety for mammalian erythrocytes than AmB.

## Discussion

Due to their escalating incidence and unacceptably high mortality, invasive fungal infections have become a great challenge for human health care [40, 41]. The main constraints for IFI therapies lie in the limited number and significant adverse effects/drug interactions of available antifungal agents because fungal pathogens are eukaryotic and have a structure and metabolism similar to the mammalian hosts [42, 43]. In addition, these pathogenic fungi can adopt various strategies to counteract the antifungal effects, which subsequently leads to the emergence of drug resistance [44, 45]. Therefore, it is important to develop novel agents or therapeutic strategies to overcome these constraints.

Due to their small size and high surface-volume ratio, nano-Ag exhibit significant broad-spectrum antimicrobial activities but less toxicity to mammalian cells than traditional Ag ion agents and thus are becoming promising “nanoantibiotic” candidates [46, 47]. Recently, a novel core-shell architecture of silver nanostructure, Pd@Ag NSs, was created using deposition techniques based on palladium seeds, which imparted the nanoparticles with uniform sizes/shapes and thus enhanced their biocompatibility [33, 38]. These novel nanoparticles have displayed excellent inhibitory effects on both Gram-negative and Gram-positive bacteria [39]. In the present investigation, we examined the antifungal activity of Pd@Ag NSs against common IFI pathogens.

Here, we showed that Pd@Ag NSs with different sizes had similar antifungal effects on the same pathogen (Table 1). For each pathogen, such as *C*. *albicans*, the discrepancy of the MIC values did not exceed 4-fold among Pd@Ag NSs with different diameters. The results are not consistent with a previous report in which sliver nanomaterials exhibited a size-dependent toxicity in several organisms including *Saccharomyces cerevisiae*, and the toxicity of silver nanoparticles decreased with increasing primary particle size [48]. We speculated that this might occur due to following reasons. One important factor was the presence of the core structure Pd NSs, which was the determinant for the size of the Pd@Ag NSs. The Ag atoms were epitaxially grown and homogeneously distributed throughout the core-shell nanosheets [33, 39], and thus the Pd size might have a subtle effect on the quantity of the released Ag ions that leads to cell damage or cell death. The shape was another critical factor for the chemical properties and fungal toxicity of silver nanoparticles [49]. Due to the presence of 6 edges, the distinct hexagonal shape of Pd@Ag NSs might contribute to the fungal toxicity and thus mask the size-dependent effects.

Our study first demonstrated that Pd@Ag NSs had discrepant antifungal effects against a variety of pathogenic fungal species, in which *Cryptococcus spp*. was most susceptible, while *C*. *glabrata* and *R*. *oryzae* were relatively insensitive. It was estimated that the Ag ions released from the nanosheets could attach to the cell wall/membrane of the microorganisms and induce dose-dependent damage to the cell wall and membrane structure [28, 50]. The interspecies discrepancy in drug sensitivity might be attributed to the differences in membrane structures and the compositions of the cell wall. For example, the cell wall of *C*. *neoformans* is composed of a substantial amount of glucans and a small amount of chitin, while the major component of the cell wall in *R*. *oryzae* is chitosan/chitin [1, 51]. The fungistatic activity of Pd@Ag NSs was not associated with the molecular type or source of *Cryptococcus spp*. and was generally superior to that of FLZ and similar to that of AmB (Table 2). More importantly, Pd@Ag NSs at low concentrations also displayed a good inhibitory effect on FLZ-resistant strains, indicating its potential in treating cryptococcosis.

However, Pd@Ag NSs alone produced less efficient killing of *Cryptococcus spp*., unless the concentration was increased to 16–64-fold of the MIC_max_, indicating that this agent might be not be suitable as an anticryptococcal monotherapy. Fortunately, the assays of drug combinations revealed a significant synergism between Pd@Ag NSs and AmB against *Cryptococcus spp*. The synergistic effect significantly reduced the concentration of AmB to 0.125 μg/mL and and thus lessened its potential toxicity. It is well known that AmB is utilized as a primary fungicidal agent against *Cryptococcus* spp. in the induction therapy, and early fungicidal activity is a critical determinant for clinical outcome of cryptococcosis[52, 53]. However, the clinical application of AmB is limited largely due to its dose-related toxic effects, which include haemolytic anaemia, nephrotoxicity, and hypokalaemia [11, 54, 55]. We speculated that combination with Pd@Ag NSs might be helpful to further extend the application time of AmB, and thus enhance the early clearance of *Cryptococcus* spp. in primary infection site. Furthermore, our haemolytic activity assessment confirmed the dose-dependent toxicity of AmB on human erythrocytes, but Pd@Ag NSs displayed much better safety, similar to that of FLZ. The safety of Pd@Ag NSs at low concentrations has also been confirmed on other mammalian cells such as liver cells and HeLa cells by previous reports [33, 39]. These results clearly demonstrate that Pd@Ag NSs are a promising adjunct therapeutic agent for cryptococcosis, especially in combination with AmB.

To better understand the antifungal mode of action of Pd@Ag NSs, the morphology and membrane permeability of *C*. *neoformans* treated with Pd@Ag NSs were further investigated (Figs 3 and 4). The most significant change was the disruption of the cell integrity in treated yeast cells, which exhibited a disordered arrangement of the cell wall, separation of the cell wall from the capsule and membrane, and enhanced membrane permeability. Similar alterations in the cell wall/membrane were also observed in *C*. *neoformans* and *C*. *albicans* after treatment with nano-Ag [50, 56–58]. It has been reported that the release of Ag ions from nano-Ag could detrimentally interact with membrane-bound proteins, resulting in a loss of cellular integrity in prokaryotic systems [59]. Similar conclusive evidence is still lacking for fungal systems. Recently, a transcriptomic analysis of *S*. *cerevisiae* confirmed that nano-Ag exposure could lead to significant up-regulation of the genes that encode cell wall proteins and membrane-bound transporters, which has also been supported by the existence of sulfur-containing Ag clusters preferentially located at the cell wall periphery in *S*. *cerevisiae* [60, 61]. We speculated that Pd@Ag NSs might exploit similar mechanisms to disintegrate the cell walls and cytoplasmic membranes in *C*. *neoformans* and other fungi. Along with the disruption of cell integrity, intracellular organelles also displayed prominent alterations in cryptococcal cells exposed to Pd@Ag NSs. A dramatic decrease in free ribosome and cytoplasm density indicated the inhibition of protein synthesis by Pd@Ag NSs. Similar microstructure changes have not been reported in fungal pathogens, which might represent a novel mechanism of fungicidal activity of nano-Ag. In addition, we also found dramatic enlargement and an increase in the number of vacuoles with Pd@Ag NSs treatment. Intense vacuolation has been previously described in *C*. *albicans* exposed to nano-Ag [50]. It could be inferred that fungal cells might rely on such a survival strategy to enhance protein transportation in response to cellular stress caused by the nano-Ag. This cellular stress might also enhance the energy demand and thus result in an increase in the number of mitochondria in *C*. *neoformans*.

In summary, Pd@Ag NSs at low MICs exhibited potent antifungal activity against various strains of *C*. *neoformans* complex species, including FLZ-resistant strains, which could likely be attributed to destruction of cell integrity and disturbance of protein synthesis and energy metabolism. Furthermore, Pd@Ag NSs also exhibited strong synergistic anti-cryptococcal fungicidal effects when combined with AmB, but with much better safety on erythrocytes than AmB. Therefore, Pd@Ag NSs might represent a potential adjunctive agent for the treatment of cryptococcosis. However, the development of Pd@Ag NSs is still in the infancy of preclinical stage, and further investigations such as in vivo anticryptococcal, pharmacokinetic-pharmacodynamic, and toxicity studies are needed.

## Materials and Methods

### Chemicals and strains

The chemicals used for nanoparticle synthesis were obtained from the following corporations. Pd(acac)2 (99%) was purchased from Alfa Aesar. Poly(vinylpyrrolidone) (PVP, MW = 30000); N,N-dimethylacetamide; N,N-dimethylformamide (DMF); NaBr; and silver nitrate (AgNO_3_) were obtained from Sinopharm Chemical Reagent Co. Ltd. (Shanghai, China). N,N-dimethylpropionamide and tetrabutylammonium bromide (TBAB) were obtained from Aldrich and Acros Organics, respectively.

In this study, a total of 55 fungal strains were utilized, including 46 *Cryptococcus spp*. strains, 7 *Candida spp*. strains, 1 *Aspergillus fumigates* strain, and 1 *Rhizopus oryzae* strain (Tables 1 and 2). Most of the strains were available in our lab, while the *R*. *oryzae* strain was kindly provided by Prof. Xu Deqiang (Fudan University, Shanghai). All yeast isolates were maintained on Sabouraud dextrose media (SDA, BD-Difco, USA) or yeast peptone dextrose media (YPD, BD-Difco, USA), and the filamentous strains were cultured on potato dextrose agar media (PDA, BD-Difco, USA).

### Synthesis and characterization of Pd@Ag NSs

Pd nanosheets (Pd NSs) were synthesized as described previously with slight modification [33, 39]. In brief, 10 mL different sized Pd nanosheets solutions were treated with continuous H_2_ bubbles for 40 min. The reaction system was closed just before the remove of H_2_ gas. Then, AgNO_3_ solution was quickly injected to Pd nanosheets solution. One hour later, the reaction was stopped by venting the system. Unreacted silver ions were washing off by acetone and ethanol. Then Pd@Ag nanosheets were stored at 4°C for future use.

The transmission electron microscope (TEM) studies were performed using a TECNAI F-30 high-resolution TEM operating at 300 kV. The Pd and Ag contents were measured by inductively coupled plasma mass spectrometry (7500CE, Agilent).

### Microdilution assay for antifungal activity

The in vitro fungistatic activity for yeasts and filamentous fungi was tested using the broth microdilution method as outlined in the Clinical and Laboratory Standards Institute (CLSI) document M27-A3 and the M38-A2 guidelines, respectively[62, 63]. All microdilution assays in this study were done at least in triplicate. AmB (Sigma-Aldrich, USA) and FLZ (Sigma-Aldrich, USA) were used as controls in this study. The final concentrations were 0.125–64 μg/mL for Pd@Ag NSs and FLZ, 0.03–16 μg/mL for AmB. RPMI1640 liquid medium (GIBCO, NY, USA) was buffered to pH 7.0 with 3-(N-morpholino)propanesulfonic acid (MOPS) (Bio Basic Inc, Ontario, Canada) and used as the culture medium. Yeast strains were cultured in SDA media for 24 or 48 h, while filamentous fungi were cultured in PDA media for 7 days. The final inoculum concentration was 1–5×10^3^ cells/mL for yeasts and 0.4–5×10^4^ cells/mL for filamentous fungi. The 96-well flat-bottomed microtitration plates were incubated at 35°C and measured after incubation without agitation for 48 or 72 h. The minimal inhibitory concentration (MIC) for AmB and Pd@Ag NSs was defined as a 100% growth inhibition compared to the drug-free control well, while the MIC for FLZ was 50% growth inhibition. Checkerboard microdilution assays were also performed to evaluate the effect of the combination of AmB and Pd@Ag NSs using the model-fractional inhibitory concentration index (FICI) method [64]. The FICI was calculated with the following equation: FICI = FIC_AmB_+FIC_Pd@Ag_, where FIC is the MIC of the combination/the MIC of each drug alone. Synergy was defined as an FICI value less than 0.5, and indifference was defined an FICI value between 0.5 and 4, while antagonism was defined as an FICI value higher than 4. An FICI result between 1 and 4 indicated no interaction.

The fungicidal effect was determined by counting the CFU of the suspension from each well after it was plated onto SDA media and incubated for 48 h. The minimal fungicidal concentration (MFC) was defined as the minimal drug concentration at which at least 99% of fungal cells were killed relative to the original inoculum. The synergistic fungicidal effect between Pd@Ag NSs and AmB for *Cryptococcus spp*. was evaluated based on the fractional fungicidal concentration index (FFCI) [64]. The FFCI was calculated using the following equation: FFCI = [Pd@Ag]/MFC_Pd@Ag_+[AmB]/MFC_AmB_, where MFC_Pd@Ag_ and MFC_AmB_ are the concentrations of Pd@Ag NSs and AmB, respectively, and [Pd@Ag] and [AmB] are the concentrations at which Pd@Ag NSs and AmB, in combination, are fungicidal. FFCIs<0.5 indicate synergistic interactions, FFCIs = 0.5–4.0 indicate no interaction, and FFCIs>4.0 indicate antagonistic interactions.

### Time-killing assay

*C*. *neoformans* (H99) cultures were prepared at an initial inoculum of 10^5^ cells/mL. Different concentrations of Pd@Ag NSs (1, 2, 4, and 8 μg/mL) were added in combination with AmB (1 μg/mL) or FLZ (8 μg/mL). After incubation with agitation at 30°C for 24–72 h, a 100 μL aliquot was removed from each solution and serially diluted 10-fold in sterile water. The diluted samples were plated on SDA agar and incubated at 30°C for 72 h. The number of CFU was then determined. The experiments were performed in triplicate. Fungicidal activity was defined as a reduction of more than 3×log10 of the original inoculum. Synergism and antagonism were defined as a decrease or increase, respectively, of more than 2 ×log10 of the inoculum of the drug combination group compared to that of the Pd@Ag NSs alone group [65].

### Transmission electron microscopy

The morphologies of *C*. *neoformans* (H99) before and after the treatment with Pd@Ag NSs were observed under a JEM-1230 transmittance electron microscope (JOEL, Japan) using an acceleration voltage of 80 kV. The H99 strain was cultured to log-phase in YPD liquid medium, harvested, and incubated with 80 nm Pd@Ag NSs at a final concentration of 16 μg/mL for 24 h at 37°C. Yeast cells were harvested by centrifugation at 1600 g for 5 min and washed with PBS buffer. The samples were fixed and embedded into Spurr resin mixture using standard procedures [66, 67]. Ultrathin sections (70–90 nm) were obtained using a Leica ultramicrotome and collected on copper grids. The grids were stained with uranyl acetate and Sato’s lead and examined with a JEM-1230 TEM at 80 kV. Digital micrographs were acquired with a 16-bit Gatan Orius SC (200w) 2048x2048 CCD camera.

### Cell membrane permeability assay

The cell membrane permeability of *C*. *neoformans* (H99) was detected as described previously [68]. Briefly, H99 cells (1×10^7^ cells/mL) were incubated with 10 μM calcein acetoxymethyl ester (calcein AM, Fanbo Biochemicals, China) for 2 h at room temperature and then washed three times with PBS buffer. Pd@Ag NSs (1–128 μg/mL) were added and incubated for 3 h at 37°C, while AmB (2 μg/mL) and PBS were utilized as controls. The mean fluorescence intensity (MFI) of cellular calcein in each sample was detected and quantified by flow cytometry analysis (Maflo Astrios flow cytometer). Each experiment was repeated in triplicate.

### Haemolytic activity assessment

Human red blood cells were diluted with PBS to a concentration of 4% [69]. Then, 50 μL of Pd@Ag NSs(80 nm), AmB, or FLZ solution was mixed with 50 μL of the erythrocyte suspension at final concentration 1–128 μg/mL. The samples were incubated at 37°C for 1 h and then centrifuged for 5 min at 250 g. The optical density was measured at 655 nm. One hundred percent haemolysis was achieved with the Red Blood Cell Lysis Buffer (Beyotime, Shanghai, China).

### Statistical Analysis

The data were expressed as the means±S.D. and analysed with SPSS 18.0 statistical software. Analysis of variance (ANOVA) and Student’s *t*-test (two-tailed) were performed to determine the statistical significance between the treatment and control groups. A *P* value less than 0.05 was considered to be statistically significant.

### Ethics Statement

The erythrocyte samples were isolated from blood of healthy volunteers. All the participants were fully aware of the purpose, process and method of our study, and voluntarily joined this study with their written informed consents. This work was approved by the Committee on Ethics of Biomedicine Research, Second Military Medical University.
